# Dialogic Health Education to Reduce COVID-19 Disparities and Increase Health Literacy in Community and Correctional Settings: Protocol for a Two-Pronged Health Education Program

**DOI:** 10.2196/37713

**Published:** 2022-10-21

**Authors:** Farah Kader, Stephanie Kruchten, Marc Campo, Kimberly Collica-Cox, Charis Davidson, Adrienne Wald, Dial Hewlett Jr

**Affiliations:** 1 Westchester County Department of Health White Plains, NY United States; 2 School of Health and Natural Sciences Mercy College Dobbs Ferry, NY United States; 3 Dyson College of Arts and Sciences Pace University New York, NY United States

**Keywords:** community engagement, dialogic learning, training of trainers, COVID-19, health literacy, correctional facility health, health equity, racial disparities, community participation

## Abstract

**Background:**

COVID-19 vaccines significantly reduce rates of hospitalization and death for those infected with the SARS-CoV-2 virus. Those facing social oppression, including people of color, experience heightened risk for COVID-19 and comorbidities, but are often mistrustful of governmental agencies and initiatives, contributing to low vaccine uptake and a reluctance to access vital health care services. Dialogue-based health literacy interventions may mitigate mistrust and increase access to health services and information, subsequently increasing rates of vaccination and other behaviors that reduce COVID-19 risk.

**Objective:**

To improve health literacy and reduce COVID-19 disparities, the Westchester County Department of Health, in partnership with two universities, community- and faith-based organizations, and the Westchester County Department of Correction, co-developed a health education program for community members, correctional officers, and incarcerated jail residents in Westchester, New York. Specific objectives are to increase preventative health behaviors, positive attitudes toward use of public health protocols, full vaccination or intentions to vaccinate, health care information understanding, health provider care access, clear communication with health care providers, and personal health care decision-making.

**Methods:**

Grounded in dialogic learning, the program entails training community-based “trusted messengers” and correctional officers to lead health information sessions in community and correctional settings. During the grant period, the program intends for 80 community-based trusted messengers to receive training from the Department of Health and will be expected to reach a goal of 100 members (N=8000) of their communities. Correctional staff with experience delivering educational programs will be trained to facilitate sessions among 400 correctional facility residents and 600 correctional staff.

**Results:**

Pre-post surveys will assess changes in health behaviors, attitudes, and perceptions. The program has been administered in the correctional facility since February 2022, with information sessions expected to cease for correctional staff and residents in June 2022 and November 2022, respectively. An initial cohort of community-based trusted messengers began training in February 2022, and information sessions have been scheduled in various virtual and community settings since March 2022. As of April 2022, the two-pronged health education program has reached 439 correctional officers, 98 jail residents, and 201 community members countywide. Program evaluation findings will be released in future publications after study implementation is complete.

**Conclusions:**

Few studies have evaluated the combined effects of training-of-trainers (ToT) and dialogical learning models on behavior and health literacy. As the first known COVID-19–specific dialogue-based health education program that applies a ToT model in the community-based, correctional, and virtual settings simultaneously, this study fills a gap in current knowledge about health literacy and health behavior in marginalized populations. Thus, this evidence-based framework can remedy COVID-19 disparities while also addressing risks for a host of health-related issues at the community level, potentially serving as a best-practice model for future health programs.

**International Registered Report Identifier (IRRID):**

PRR1-10.2196/37713

## Introduction

### Background

Individuals infected with SARS-CoV-2, the virus that causes COVID-19, are significantly less likely to experience death or severe disease when vaccinated [1]. Despite vaccine availability and calls for population-wide COVID-19 vaccination from health care professionals and public health agencies, many individuals in the United States remain unvaccinated [2,3]. Further, groups experiencing various levels of social oppression are not only at heightened risk of COVID-19 morbidity and mortality but are also less likely to receive a COVID-19 vaccine than the population as a whole [4-6].

Sociodemographic characteristics correlated with high COVID-19 positivity at the zip-code level include high percentages of people of color, limited English language proficiency, household crowding, and poverty [7-9]. Reasons for these associations are multidimensional; for example, inequities related to poverty and structural racism can exacerbate comorbidities, thereby increasing risks of COVID-19 severity [10-12]. Moreover, a lack of culturally and linguistically appropriate health communication can thwart contact with the health care system and complicate discernment of reliable health information, such as COVID-19 prevention recommendations [13,14].

Social factors also correspond to low COVID-19 vaccine uptake [15]. Race, ethnicity, and language are linked with vaccine refusal and reduced patient-provider interaction, often due to past experiences of medical discrimination [16,17]. As a result, people of color in the United States are more likely to mistrust health authorities, resulting in lower COVID-19 vaccination rates than the national average [2,18].

Incarcerated individuals face added dimensions of social vulnerability and COVID-19 disparities due to crowding, poor sanitation, and lack of personal protective equipment [19,20]. Although much of the public focus during the pandemic was on state and federal prison systems, with little attention paid to county jails, the jail’s transient population creates a disproportionate risk for COVID-19 transmission. This is because new arrests bring infection from the community into the jail, while prisons receive new admissions from a medically cleared jail population [21]. These factors contribute to heightened COVID-19–related risks to the staff and residents of the county jail [22,23].

Despite this disparity, both correctional facility staff and residents demonstrate disproportionately low rates of COVID-19 vaccine uptake [24]. Mistrust toward correctional staff, combined with limited health education in correctional facility settings, can contribute to vaccine skepticism among the incarcerated [25]. In addition, correctional officers more frequently demonstrate vaccine refusal than the population as a whole, with some citing distrust of correctional administrators, beliefs in conspiracy theories, or other misinformation about vaccines [24]. Medical mistrust among both correctional officers and residents may inhibit access, use, and outcomes of health-related interventions that aim to increase vaccination rates.

### Advancing Health Literacy

These disparities arise from complex issues occurring over long periods of time; as such, addressing these issues at a population level requires long-term structural solutions [26]. However, health literacy interventions may help to meet more immediate health needs among the underserved. The US Department of Health and Human Services (HHS) National Action Plan to Improve Health Literacy uses a definition of health literacy that underscores health care access, health information understanding, and informed health decision–making [27]. This definition guides the HHS Office of Minority Health’s initiative Advancing Health Literacy to Enhance Equitable Community Responses to COVID-19 (AHL). AHL grants fund programs that “demonstrate the effectiveness of working with local community-based organizations to develop health literacy plans to increase the availability, acceptability, and use of COVID-19 public health information and services by racial and ethnic minority populations” [28].

This protocol outlines the guiding principles and methods used to implement an AHL-funded health education program and evaluation of its effectiveness among priority populations in Westchester County, New York.

### Theoretical Approach

Educational programs that integrate dialogic learning approaches can give power to learners and acknowledge the value of their experiences and cultures, creating more equitable conditions for sharing information and providing different points of view [29,30]. Studies have shown that discussion-based learning can be more effective in improving health and motivating self-education than a “banking” method of education, which views students as empty vessels to be filled by teachers [29-31].

The program also applies a training-of-trainers (ToT) framework to equip select individuals with the tools and information necessary to facilitate dialogic health information sessions in various settings. Those selected, referred to as “trusted messengers,” will be individuals who are considered trustworthy in their communities and can lead culturally sensitive, linguistically appropriate COVID-19–focused information sessions in either correctional facilities or community-based settings. Studies have found that enhanced trust between teachers and learners can encourage program participants to engage in constructive dialogue, ask questions that enhance understanding, and put health information into practice [29,32-34].

Yet, perceived health expertise is also shown to be important for influencing health behavior and enhancing trust [35,36]. A recent study of vaccine hesitancy among Black Americans found that “medical professionals they were familiar with and trusted could influence their decision to take a COVID-19 vaccine” [35]. Thus, the proposed program engages a team of health professionals who may offer support to trusted messengers who lack prior experience with health education or health care delivery.

### Health Education in Correctional Facilities

Health education programs led by trusted individuals in correctional facilities have improved behavior intention and attitudes toward risks related to HIV, tuberculosis, and hepatitis C viral infection [37-40]. This suggests that training those who may be perceived as trusted messengers for jail residents can be equally effective for affecting the same outcomes (attitudes and intended behaviors) for COVID-19–related risks.

Lessons learned from previous dialogic correctional facility–based HIV programs indicate that “inmate questions and ensuing discussions are an important training ground for applied prevention” [41]. Dialogic education is particularly important in correctional environments, where staff and residents may frequently feel scrutinized, as opening communication between teachers and learners can serve as a rare avenue of personal control and improve individual health decision–making as a result [42].

### Overview of the Know Better, Live Better Program

The Westchester County Department of Health (DOH) has designed an AHL-funded program that applies ToT and dialogic learning models of education to correctional officers, jail residents, and community members in geographic areas of focus, relying on trusted messengers to lead health information sessions. Trusted messengers receive training on health information delivery and a semistructured lesson plan for leading a 1-hour information session. The sessions themselves intend to be discussion-based, allowing a bidirectional transmission of information between trusted messengers and program participants.

**Figure 1:**
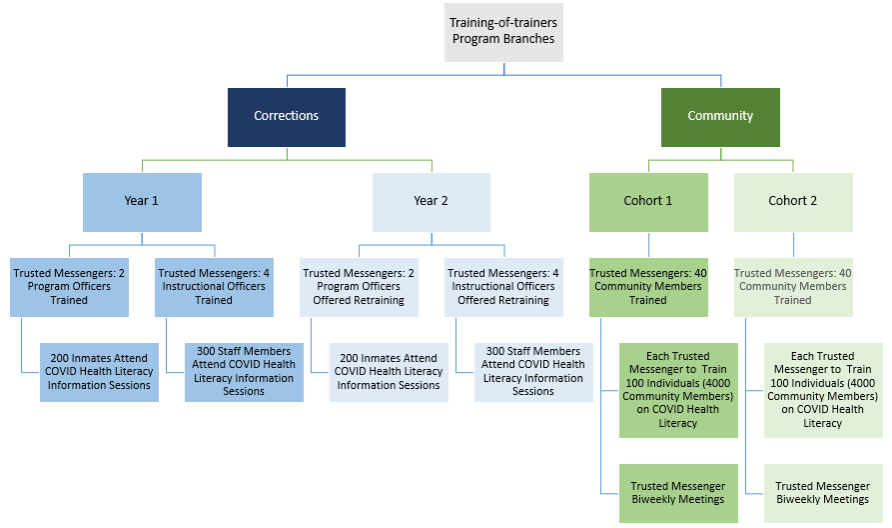
Overview of Know Better, Live Better program branches, and anticipated reach of community-based and correctional facility–based programs.

There are two parallel branches of this program, entitled Know Better, Live Better (KBLB). The first is based in community settings, engaging community- and faith-based organizations (CBOs), and focusing recruitment efforts in neighborhoods with high social vulnerability and COVID-19–related risk. The second branch partners with the Westchester County Department of Correction (DOC) to address health literacy and COVID-19 among correctional officers and jail residents. Figure 1 illustrates the number of trusted messengers participating in ToT and the expected study population per branch.

### Goals and Objectives

We hypothesize that a dialogic learning program, co-developed by CBO partners, will help achieve the overall goals to improve health literacy and reduce COVID-19–related racial and ethnic disparities in Westchester County. After analyzing pre-post survey changes among participants of each information session, we expect increases in preventative health behaviors, positive attitudes toward use of public health protocols, full vaccination or intentions to vaccinate, health care information understanding, health provider care access, clear communication with health care providers, and personal health care decision–making. We will also use demographic questionnaires to gauge any socioeconomic factors mediating program effectiveness, as measured by these objectives.

## Methods

### Community-Based Program Design

#### Overview

We aim to train approximately 80 trusted messengers to reach a goal of 8000 individuals in the geographic area of focus by the end of December 2022. These areas were selected using the Centers for Disease Control and Prevention Social Vulnerability Index (SVI), which encompasses 4 key domains: socioeconomic status, housing and transportation, minority status and language, and household composition. Program staff identified 55 census tracts ranked with the highest social vulnerability in the county. These census tracts overlap with zip codes with high COVID-19–related morbidity and low vaccine uptake; thus, they constitute the geographic area of focus for this project. These are the areas in which program staff will concentrate efforts to recruit all information session participants, trusted messengers, and CBO partners. However, zip code of residence will not be considered among the exclusion criteria for any partner, trusted messenger, or participant to ensure that program benefits are wide-reaching. As of April 2022, 26 trusted messengers completed training and collectively reached 201 community-based participants.

#### Training of Trainers

All trusted messengers will be screened through an online form and interviews with DOH staff. Applicants must be at least 18 years old, have access to a computer with internet connection, and have sufficient availability to deliver the program to at least 100 participants in geographic areas of focus. Trusted messengers are also required to be vaccinated for COVID-19 before any health information sessions begin. Preference will be given to bilingual trusted messengers who are comfortable giving presentations in other languages, as well as those with strong connections in Westchester communities. Each trusted messenger will receive a stipend for completing required program activities.

Program staff facilitate 6-hour trainings spread over 1 week to provide trusted messengers with information to prepare them for discussion facilitation. This includes COVID-19–related health information, skills related to dialogic learning, and the curriculum outline. Each trusted messenger receives a handbook that contains this information in written form. Trusted messengers will participate in biweekly, 1-hour check-in meetings with program staff to provide opportunities to discuss challenges they have incurred.

#### Study Settings

Community-based information sessions are designed to be either virtual or in person, in order to reach those with limited internet access, and will be limited to 30 individuals to foster productive and engaging discussions [43,44]. Partnering CBOs may host in-person sessions so that attendees can engage with health information in familiar community settings such as churches and cultural centers. Virtual sessions will be hosted through the Cisco WebEx video conferencing system with a DOH account.

#### Health Information Session Development

The proposed information sessions cover issues related to COVID-19, such as disease prevention measures and barriers to vaccine acceptance. These topics are contextualized in a broader discussion about health literacy, which includes accessing health care, understanding health information, and making individual health decisions. Trusted messengers will be provided with standardized resources that are necessary for program delivery, including presentation slides and handouts. The information sessions will be piloted with academic partners and CBOs to ensure appropriateness, applicability, and quality.

#### Participant Recruitment

CBOs and DOH program staff will assist trusted messengers with recruitment and locating meeting spaces for in-person sessions. Trusted messengers and/or CBOs are to ensure that recruitment efforts are made before the scheduled session. Trusted messengers and/or CBOs will make initial contact with the participants, depending on who has identified them as an interested participant.

For virtual sessions, program staff will provide trusted messengers with a WebEx video conferencing link to share with their intended audience. Registration links will also be made publicly available on the program website. In-person participants may learn about scheduled sessions in any language through personal referral, public notice, and other promotional efforts that DOH program staff and partners make.

#### Health Care Professional Support Team

Because health care professionals who are trusted in their communities can effectively influence health perceptions and behavior, a support team comprised mainly of DOH-employed registered nurses and nurse practitioners will be present for all sessions (virtual and in person) as an additional source of support for trusted messengers who lack health expertise.

#### Data Collection

**Figure 2:**
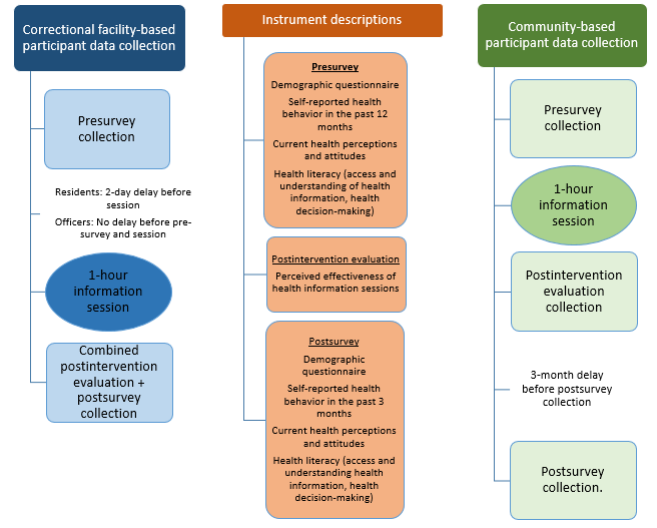
Data collection time points and instruments used in each program branch.

Data will be collected during this project using multiple instruments, as outlined in Figure 2. First, a presurvey will be distributed prior each information session. This questionnaire is designed to assess knowledge, attitudes, and behaviors regarding COVID-19 protocols and vaccines. There will also be questions related to health literacy, such as participants’ ability to navigate the health care system, discern reliable health information, and use health information to make decisions. The included questions were adapted from previously validated tools: European Health Literacy Survey (HLS-EU) Questionnaire and the Understanding America Study (UAS230) [45,46]. Demographic items will include race/ethnicity, gender, age group, marital status, and employment.

Immediately following the information session, program staff will distribute a short questionnaire (“postintervention evaluation”) that asks participants to assess the perceived efficacy of the 1-hour session. The questionnaire consists of 7 Likert items asking whether the session had impact in terms of health information understanding, health information access, and health care decision–making.

Participants in the in-person information sessions will be offered paper surveys. Virtual program participants will complete the surveys from an open link from Research Electronic Data Capture (REDCap), a secure, web-based application designed to support data capture for research studies [47]. In-person participants will receive paper versions that will be collected by select program staff and entered manually into REDCap.

In addition to collecting data through surveys, program staff will be present at each information session to track general information about the session logistics. Staff will also use an observation guide to document the broad themes that arise during the facilitated discussions, providing a source of qualitative data.

#### Supplementary Data Collection

DOH research staff will assess the broader public health influence of the AHL program by monitoring public health surveillance data at the zip-code level, comparing changes in geographic areas of focus to the rest of the county throughout the grant period. These data include COVID-19 positivity rates, calculated using test results reported to the New York State Electronic Clinical Laboratory Reporting System. Data from the New York State Immunization Information System will also track vaccine registration rates across zip codes in the county. Staff will take into account any national, state, or local policy changes, and other potential reasons for changes in COVID-19 positivity and vaccination rates.

To evaluate the effect of the AHL program on primary care access, staff will compile a list of primary care providers located in geographic areas of focus. They will request information from a subset of providers on new patient visits and service utilization throughout the grant period on a monthly basis.

#### Incentives

The use of incentives in research encourages innovation, provides motivation, and promotes strategic decision-making [48]. Evidence-based incentivization through nonmonetary means is linked to increased data sharing, specifically when individuals are provided with a badge or certificate [49]. Therefore, all participants will receive a certificate of completion for attending the health information session.

Individuals receiving a financial incentive for immunization were three times more likely to become immunized compared to those not receiving an incentive [50]. Thus, to encourage program participation, individuals will be provided a US $10 gift card incentive as compensation for their time spent at the session. A US $10 electronic gift card will also be provided to those who complete the optional 3-month postintervention survey.

#### Research Ethics and Approval

The materials and methods of the community-based portion of the project were reviewed and approved by the Mercy College Institutional Review Board (IRB; Project #21-82) on February 8, 2022.

### Correctional Facility–Based Program Design

#### Overview

DOH staff will train program officers and instructional officers to educate 400 correctional facility residents and 600 correctional staff, respectively, during the 2-year grant period. While the officers will lead information sessions, DOH program staff will attend to provide administrative assistance and complete informed consent. The training and 1-hour lesson plans will closely resemble those designed for the community-based branch of the AHL program, with further tailoring to meet audience-specific needs, including COVID-19 protocols and special health care resources. As of April 2022, the correctional facility–based education program has reached 439 correctional officers and 98 jail residents.

In the correctional facility, there are two sets of discussion leaders, who play the same role as trusted messengers in community-based sessions. Program officers with experience delivering health programs to jail resident comprise the first group. Although they are uniformed officers, the relationships they have with residents are more positive than those of other officers, owing to their roles as program leaders. The second group consists of correctional officers who typically act as instructors for fellow officers during annual training days. Thus, the two sets of learners for the correctional facility–based program are residents and correctional officers. All discussion leaders will be required to be vaccinated for COVID-19.

#### Training of Trainers

Program staff will provide approximately 6 hours of training to both program and instructional officers. Training sessions will cover COVID-19–related information, AHL program goals, informed consent, and advice for moderating discussions among residents and officers. Like community-based trusted messengers, program and instructional officers will receive necessary resources, including a trainer manual, pilot session recordings, and access to medical support via video conference during the information sessions for any health questions.

#### Study Settings

All correctional facility–based sessions will occur in person at a Westchester County–based jail. The jail consists of the Jail and Penitentiary Divisions. The Jail Division houses individuals aged 18 years and older, including men and women awaiting sentencing and women sentenced to terms of 1 year or less. The Penitentiary Division houses men aged 18 years and older sentenced to terms of 1 year or less. The DOC operates the jail and is accredited by the American Correctional Association with the medical operations accredited by the National Commission on Correctional Health Care.

Health information sessions for correctional staff will be held in a training academy classroom during annually scheduled training days. Participating staff members will be asked to voluntarily complete a presurvey immediately before the information session and a postsurvey immediately afterward. A program staff member and a member of the health care professional support team will be virtually present at all sessions.

Health information sessions for residents will take place in the housing units’ recreational rooms. Due to COVID-19, there is an anticomingling policy between housing units. Therefore, the program will be offered to each unit individually to ensure complete jail coverage and minimize disease spread. Per DOC request, all residents who would like to participate will be allowed to attend with potential discussion group sizes of up to 40 individuals.

#### Health Information Session Planning

The correctional facility–based curriculum, after undergoing CBO partner review, will be tailored to address needs and specific concerns for officers and residents based on recent literature. These tailored versions will be reviewed by a health educator and members of DOC leadership, each with experience delivering programs in correctional facilities. Residents will also receive a list of local health care providers who can be accessed upon release.

#### Participant Recruitment

Correctional officers will participate in KBLB health information sessions as part of their annual “pay back” days, dedicated for training purposes. DOC leadership has dedicated 1 hour of open training time to the KBLB program to increase health literacy and address COVID-19 vaccine resistance among staff.

To recruit residents, program officers will provide information about the KBLB program to residents ahead of the scheduled lessons. Per DOC protocols, residents must write their name on a sign-up sheet if they are interested in participating so that the officers may account for all residents during scheduled session times. This also ensures that participants receive their incentive, voluntary presurvey, and informed consent.

#### Data Collection

Data will be collected during the correctional facility–based component of this program using two instruments: the “presurvey” and the “postsurvey.” The presurvey will be distributed prior to the discussion sessions, while the postsurvey will be distributed immediately following the discussion session. Neither is required to participate in the discussion. These surveys are based on the same instruments that were used for the community-based education program (Figure 2).

Unlike the community-based branch, the correctional facility branch of the program will combine the 3-month postsurvey with the postdiscussion evaluation. The postsurvey is identical to the presurvey, including all Likert scales, so that pre-post changes can be accurately assessed. Open-ended questions will be used to understand primary concerns regarding COVID-19 vaccines and other public health prevention recommendations before and after the sessions. All surveys will be paper-based and responses will be manually entered into REDCap.

Like the community-based component, program staff will complete an observation guide detailing the number of participants in attendance and documenting characteristics of the correctional facility–based discussion session. Staff will record common themes discussed during the sessions, unique features of each session, questions that arise, and discussion topics. The observational guide will include both qualitative and quantitative elements to capture a more complete and detailed depiction of each session.

#### Incentives

Correctional staff will participate in the program during work hours. They will receive either breakfast or lunch during the session in addition to their regular hourly pay.

Incentives for residents will be a US $10 voucher to the jail commissary for food and beverages, or a US $10 prepaid phone card. All participants will receive an incentive regardless of whether they complete surveys.

#### Research Ethics and Approval

The materials and methods of the correctional facility–based portion of the project were reviewed and approved by the Pace University IRB (Project #1843825-2) on February 11, 2022.

## Results

### Analysis of Community-Based Program Data

All survey data will be analyzed with descriptive statistics. Investigators will use *t*-tests and *χ*^2^ analyses for pre-post discussion session changes and a multivariable regression analysis to adjust for covariates such as race, ethnicity, age, and other demographic variables.

Pre- and postsurveys do not include person identifiers and subsequently will not be matched individually. However, as they will be given to a correlated group of individuals, the mean of the pretest variables will be used as a threshold score for the comparison of the post-test variables using a one-sample *t*-test. The postsurvey scores will be compared to the individual mean score of the presurvey. The surveys will be analyzed for changes in behavior or behavior intentions, attitudes, and perceptions with the following potential mediator-moderator domains: gender, age, race, ethnicity, zip code, education level, and marital status.

For item nonresponse, response rates for each question will be stratified by demographic factors to ensure systematic bias is not present due to nonrandom missing variables. If variables are found to be missing at random, imputation of items will occur.

The qualitative components of the observational guides completed by program staff will be analyzed for emergent themes using a data-driven thematic coding scheme that will be iteratively developed by the analytic team in accordance with grounded theory analysis [51,52].

Census tracts comprising the geographic areas of focus, as determined by the SVI, will be mapped to overlapping zip codes. Population counts from the US Census Bureau and data extracted from disease reporting systems will allow for zip code–level computation of outcome rates, vaccination registration rates, and COVID-19 positivity rates. To quantify disparities between zip codes that fall within geographic areas of focus and the rest of the county, a time-trend analysis will be performed, using outcome rates calculated monthly over the course of the 2-year grant period. This will allow assessment of whether changes in outcome rates in the geographic areas of focus correspond to changes in the number of AHL program sessions collectively occurring in those zip codes in a given month, while also comparing rate changes to county zip codes in which information sessions did not take place.

### Analysis of Correctional Facility–Based Program Data

The analysis plan for pre- and postsurvey changes and adjustment for covariates will not differ from the analyses performed on community-based surveys detailed above. In addition, the observation guides used for observing correctional facility–based information sessions will not differ from those guides applied to community-based sessions. Therefore, the analysis of all observation guides will also adhere to the same procedures. Analysis of open-ended responses on pre- and postsurveys will follow similar thematic analysis to the qualitative components of the observation guides.

## Discussion

### Projected Significance

While there is a body of literature examining sociodemographic predictors of poor health literacy, few studies integrate peer support and dialogic learning models, two evidence-based frameworks that can have sustainable effects on health behavior and knowledge, with COVID-19–focused health education. To our knowledge, this is the first COVID-19–specific dialogue-based education program that applies a ToT model in community-based, virtual, or correctional settings to undergo longitudinal evaluation over multiple months. We hypothesize that pre-and postsurvey analyses for all participants in all settings will demonstrate an increase in the following key outcomes: preventative health behaviors, positive attitudes toward use of public health protocols, full vaccination or intentions to vaccinate, health care information understanding, health provider care access, clear communication with health care providers, and personal health care decision–making improvement.

### Expected Benefits

In addition to meeting our outcome objectives, we anticipate that the AHL-funded KBLB program will have several benefits for both community- and correctional facility–based participants. First, engaging CBO partners at multiple stages of KBLB planning and implementation can make participant recruitment efforts more equitable, allow community perspectives to inform an educational approach, and help develop sustainable partnerships that sustain program effectiveness [53,54].

Trusted messengers can foster a learning environment in which participants feel comfortable being candid and relaying questions or concerns. For attendees with minimal contact with the health care system, access to a member of the health care professional support team may be a rare opportunity to interact with health experts in a setting that is less intimidating than a clinic or private physician practice.

The emphasis on dialogic learning also allows attendees to discuss health concerns about COVID-19, discerning reliable health information, and other issues directly to a trustworthy peer. Attendees at information sessions are also encouraged to share knowledge about barriers to health improvement and health care access in their respective communities. The information exchanged during facilitated discussions is expected to illuminate underlying reasons for health-related attitudes and/or behaviors among diverse Westchester residents who, by virtue of geographic location in high-risk zip codes, experience varying levels of social oppression.

By having both trusted messengers and health care professionals present at each of the health information sessions, the program may increase the likelihood that participants will share messages learned. Throughout the pandemic, individuals reported stronger intentions to share messages from leaders compared to citizens [55]. Therefore, the trusted messenger framework is used to encourage active participation to create a more dynamic and interactive learning environment, while the health care professional lends credibility to all information shared.

### Challenges

Potential obstacles must be considered during program implementation and evaluation. Studies have shown that dialogic learning approaches are highly dependent on the attitudes of instructors and the physical environment in which learning takes place [56]. Program staff must use the trusted messenger recruitment process to carefully vet individuals who are invested in collaborative learning, as well as those who are trusted and well-connected in the geographic areas of focus. Because certain issues related to the COVID-19 pandemic may be contentious, such as vaccination mandates, fear of being judged or criticized may hinder participation. Furthermore, information sessions held over a virtual conferencing system may pose an additional barrier to participant interaction. Thus, the ToT must emphasize discussion facilitation strategies that encourage participation and promote a safe, inclusive environment for discussion [57,58].

It will be difficult to recruit a cohort of community-based trusted messengers that is entirely representative of a diverse county population. Hard-to-reach populations may include individuals who prefer languages other than English and Spanish, individuals with disabilities, the deaf community, the LGBTQ community, elderly persons, and others. Without participating trusted messengers, CBOs, or program staff who have existing relationships with these subpopulations, participation recruitment may lack breadth; as a result, health information sessions may lack critical perspectives.

Health departments and other agencies that coordinate community-based participatory health interventions may encounter administrative obstacles during program planning and implementation phases. For instance, ToT models and CBO partnerships generally require formal agreements between funders, awardees, and partners. Grassroots organizations lacking legal departments and other administrative support may not have the capacity to properly vet proposed agreements, afford liability insurance, or provide other types of documentation that government agencies may require. Similar challenges are present for informal groups and associations that are organized by community members rather than formal institutions. Such barriers can slow down onboarding processes and delay program implementation. Monthly CBO workgroups may act as resource-sharing opportunities for large, well-equipped organizations to assist more resource-limited groups.

Some challenges specific to the correctional facility setting are related to special restrictions and policies that may interfere with best practices for health program delivery. For example, due to the COVID-19–related anticomingling policy, informational sessions for residents must take place in housing units of 35 to 40 people. Depending on the number of voluntary participants per housing unit, discussion group sizes may exceed the recommended maximum size of 30 learners per unit. To ensure that quality of the discussion is maintained, program officers who are experienced in leading larger discussions among residents will lead health information sessions. Similarly, correctional officers leading discussions for fellow officers are accustomed to instructing large groups in this setting. Finally, because the rate of vaccine uptake among correctional officers is generally low, it is a challenge to recruit discussion leaders who support vaccination and other health measures that are emphasized in the program curriculum.

Despite these challenges, program implementation may have lasting effects that result in long-term health improvement and subsequent cost savings. For example, establishing trust and understanding can aid effective health communication and resource-sharing in future health emergencies. In addition, investing in programs that motivate health literacy skills through dialogue can lead to more preventive health behaviors at the population level, reducing the potential costs associated with emergency services utilization [59]. Findings from this study will be used to make recommendations and develop best practices for future health programs.

## Appendix

Multimedia Appendix 1 Externally reviewed by: Office of Minority Health, United States Department of Health and Human Services.

## Abbreviations

AHL: Advancing Health Literacy to Enhance Equitable Community Responses to COVID-19
CBO: community- and faith-based organization
DOC: Department of Correction
DOH: Department of Health
HHS: US Department of Health and Human Services
HLS-EU: European Health Literacy Survey
IRB: Institutional Review Board
KBLB: Know Better, Live Better
OMH: Office of Minority Health
REDCap: Research Electronic Data Capture
SVI: social vulnerability index
ToT: training of trainers
