# Supplementary material for: Dialogic Health Education to Reduce COVID-19 Disparities and Increase Health Literacy in Community and Correctional Settings: Protocol for a Two-Pronged Health Education Program
Source: JMIR Res Protoc. 2022 Oct 21;11(10):e37713. doi: 10.2196/37713 (PMC9591706; doi:10.2196/37713)
Supplement: Multimedia Appendix 1 [file resprot_v11i10e37713_app1.pdf]

Advancing Health Literacy to Enhance Equitable Community Responses to COVID-19

Compilation

Application Number: CPI2021003379
Application Name: Westchester County Department of Health
State: NY   City: White Plains

Scoring Criteria

Non-Scoring Criteria

Criterion 1: Statement of Need: How well does the applicant describe the problem and contributing factors of the problem to demonstrate need for the project?

Strength:

None

Weakness:

None

General:

None

Criterion 2: Statement of Need: How well is the population(s) and geographic area of focus described using data on demographic characteristics (e.g., race/ethnicity, rural/urban, limited English proficiency) and characteristics associated with social vulnerability and individuals at highest risk for experiencing disparities in COVID-19 outcomes (e.g., COVID-19 positivity per capita rate, CDC SVI index rating)?

Strength:

None

Weakness:

None

General:

None

Criterion 3: Statement of Need: How well does the applicant demonstrate understanding of disproportionate impact of COVID-19 on high-risk and underserved racial and ethnic minority populations in the geographic area of focus?

Strength:

None

Weakness:

None

General:

None

Criterion 4: Statement of Need: How well does the applicant describe the approach for developing a disparity impact statement?

Strength:

None

Weakness:

None

General:

None

Criterion 5: Statement of Need Overall

Strength:

Page: 16-17

The applicant clearly describes the need and the scope of the problem that will be addressed by the proposed project including, the populations and geographic area of Westchester County, NY. The applicant includes charts which clearly illustrate the disproportionate impact of COVID- 19 on racial and ethnic minority populations in the geographic area of focus which show a positive correlation between COVID-19 case rates and percentage of household crowding, Black population, and people of color, English language-limited households, and poverty.

Weakness:

Page: No Page

The applicant does not describe if it developed a disparity impact statement for the proposed project.

General:

None

Criterion 6: Proposed Approach: How well does the applicant describe and discuss project details, including the project goals, objectives, and proposed outcomes?

Strength:

None

Weakness:

None

General:

None

Criterion 7: Proposed Approach: How feasible, data-driven and culturally and linguistically appropriate is the proposed approach for applying health literacy strategies to improve the access, use and outcomes of COVID-19 health information and services for the population of focus?

Strength:

None

Weakness:

None

General:

None

Criterion 8: Proposed Approach: How well does the applicant describe the approach for implementing health literacy strategies to address the Health People objectives HC/HIT-01, HC/HIT-02 and HC/HIT-03?

Strength:

None

Weakness:

None

General:

None

Criterion 9: Proposed Approach: To what extent are the proposed outcome(s) feasible, measurable and unambiguously aligned with the program goals and objectives?

Strength:

None

Weakness:

None

General:

None

Criterion 10: Proposed Approach: How well does the applicant describe partnerships with community based organizations to support the development and implementation of the health literacy plan and sustainability plan?

Strength:

None

Weakness:

None

General:

None

Criterion 11: Proposed Approach: How well does the applicant describe an adequate quality improvement approach to refine interventions and support improved health literacy related to the access, use and outcomes of COVID-19 health information and services for the populations in the geographic area of focus?

Strength:

None

Weakness:

None

General:

None

Criterion 12: Proposed Approach: How adequate and feasible is the evaluation approach for determining whether the health literacy intervention was implemented in adherence with the National CLAS Standards, whether it reached its target population described in the Disparity Impact Statement, and whether there were any changes in access, use and outcomes of COVID-19 vaccination, testing, and related activities (e.g., contact tracing, preventive behaviors)?

Strength:

None

Weakness:

None

General:

None

Criterion 13: Proposed Approach: How adequately does the applicant describe the use of data, stratified by demographic characteristics, to advance Healthy People 2030 objectives HC/HIT-01, HC/HIT-02, HC/HIT-03 and IID-D02?

Strength:

None

Weakness:

None

General:

None

Criterion 14: Proposed Approach Overall

Strength:

Page: 17-20, Appendix: 54-55

The applicant provides measurable goals and objectives along with descriptions of the evaluation approach that will meet the CLAS standards along with the numbers of trainers and participants planned to complete the proposed project. To achieve the goals and objectives the applicant explains it will use a two pronged approach to a train-the-trainer program: one branch is community-based and engages community- and faith-based organizations (CBFOs), the other involves a partnership with the Westchester County Department of Corrections (DOC). The applicant includes a chart of its partners with each of their roles and responsibilities, contributions and resources for the project. The applicant states the Minority Serving Institution, Mercy College, will lead process evaluation and make quality improvement recommendations during the project to ensure target populations are reached and proposed outcomes are being adequately measured.

Weakness:

Page: No Page

The applicant does not specifically address how it will implement health literacy strategies to address the Health People objectives HC/HIT-01, HC/HIT-02 and HC/HIT-03 in the proposed project

General:

None

Criterion 15: Organizational Capacity: How well does the applicant demonstrate a strategy for minimizing start-up delays?

Strength:

None

Weakness:

None

General:

None

Criterion 16: Organizational Capacity: How adequate are the roles and responsibilities of project leadership in providing project management and oversight?

Strength:

None

Weakness:

None

General:

None

Criterion 17: Organizational Capacity: How well does the organizational chart demonstrate adequate project staffing?

Strength:

None

Weakness:

None

General:

None

Criterion 18: Organizational Capacity: How well does the applicant describe the partnership with an institution/organization for quality improvement and program evaluation?

Strength:

None

Weakness:

None

General:

None

Criterion 19: Organizational Capacity Overall

Strength:

Page: 13-14, 48-49, 50-52

The applicant explains it is a full-service public health department with a workforce of approximately 250 people and serves a population of nearly 1 million. The applicant identifies the Evaluation and quality improvement activities will be assigned to the MSI, Mercy College. The applicant presents an appropriate project leadership for the management and oversight or the proposed project. The applicant explains it has received funding from Bloomberg Philanthropies to hire a six-member team to solely support COVID-19 operations and these staff will aid the operations of the proposed project. The applicant has included summary BIOs for the key project staff along with an organizational chart for the proposed project.

Weakness:

None

General:

None

Criterion 20: Please make your final recommendation with the understanding that Statement of Need, Proposal Approach, and Organizational Capacity are of equal importance.

Strength:

Page: 13-20, Appendix

The applicant clearly describes the proposed project, the participants and the partners to be utilized in the project which will align with the NOFO and will improve adherence to COVID-19 public health practices with high-risk and underserved racial and ethnic minority populations in Westchester County, NY.

Weakness:

None

General:

None
